# Transcranial electric stimulation for the investigation of speech perception and comprehension

**DOI:** 10.1080/23273798.2016.1247970

**Published:** 2016-11-01

**Authors:** Benedikt Zoefel, Matthew H. Davis

**Affiliations:** ^a^ MRC Cognition and Brain Sciences Unit, Cambridge, UK

**Keywords:** tES, tACS, tDCS, speech, perception

## Abstract

Transcranial electric stimulation (tES), comprising transcranial direct current stimulation (tDCS) and transcranial alternating current stimulation (tACS), involves applying weak electrical current to the scalp, which can be used to modulate membrane potentials and thereby modify neural activity. Critically, behavioural or perceptual consequences of this modulation provide evidence for a *causal* role of neural activity in the stimulated brain region for the observed outcome. We present tES as a tool for the investigation of which neural responses are necessary for successful speech perception and comprehension. We summarise existing studies, along with challenges that need to be overcome, potential solutions, and future directions. We conclude that, although standardised stimulation parameters still need to be established, tES is a promising tool for revealing the neural basis of speech processing. Future research can use this method to explore the causal role of brain regions and neural processes for the perception and comprehension of speech.

## Introduction

In imaging research on speech perception and comprehension, the independent variable is commonly the experimental stimuli that participants hear and the tasks that they perform during data collection. The dependent measure recorded is the observed brain activity “caused” by listening and/or performing these tasks (Weber & Thompson-Schill, 2010). Depending on the imaging measure used, we can therefore conclude that speech perception or comprehension is associated with activity in certain brain regions (Rodd, Davis, & Johnsrude, 2004; Silbert, Honey, Simony, Poeppel, & Hasson, 2014) or leads to neural oscillations entrained to connected speech in multi-speaker scenarios (Zion Golumbic et al., 2013; Zoefel & VanRullen, 2015c). Importantly, however, these methods do not provide evidence that these neural correlates of speech processing are necessary – or causal – for successful speech perception and comprehension: only if brain activity is manipulated or controlled – as an independent variable – are we able to deduce causal relationships between neural responses and the observed processing outcome (e.g. that activity in a given brain region or speech “tracking” by neural oscillations is necessary for speech comprehension).

Brain stimulation methods have an important role to play in establishing causal mechanisms for speech processing as illustrated in the following quote:

When the electrode was applied to the speech cortex, it did not cause a man to speak. It seemed at first to have no effect. But if the patient tried to speak while the electrode was in place, he discovered to his astonishment (and to ours at first) that he could not find his words.

This observation of the effects of direct electrical stimulation of the brain during awake neurosurgery by Penfield (1965, p. 790) is a striking demonstration of the causal role of neural activity in speech production: if this activity is disturbed, speech production is impossible. In the subsequent 50 years, electrical stimulation has become a common method for mapping of “eloquent” (language relevant) cortex during neurosurgery so as to avoid the most debilitating effects of surgical lesions (Duffau et al., 1999). This illustrates how causal brain processes for perception or behaviour can be studied using permanent lesions (Wilson, 2017) or transient brain stimulation as in Penfield (1965). Importantly, various tools have been developed within the last decades that enable the selective manipulation of neural processing without surgery. A common method for this purpose is transcranial magnetic stimulation (tMS; Adank, Nuttall, & Kennedy-Higgins, in press) while transcranial electrical stimulation (tES, an umbrella term that refers to both transcranial direct current stimulation, tDCS, and transcranial alternating current stimulation, tACS), a lesser-known tool, is increasingly attracting researchers’ attention (Herrmann, Rach, Neuling, & Strüber, 2013; Woods et al., 2016). tES has several benefits over other brain stimulation methods: (1) physical sensations due to stimulation are relatively weak (unlike the painful muscle twitches that can be induced by tMS), (2) neuronal processing is modulated rather than interrupted by producing irrelevant neural activity (see below), permitting the use of experimental designs in which tES is used to enhance rather than disrupt processing, and (3) tES unlike tMS is completely silent. These factors make tES a valuable tool for speech research, and further suggest exciting possibilities for clinical applications of this method.

## Historical background

Already in the eighteenth century (and potentially even earlier; Priori, 2003), researchers such as Luigi Galvani, Alessandro Volta, and Giovanni Aldini experimented by applying weak current to the bodies of humans and other animals (for reviews, including references to the original literature, see Parent, 2004; Priori, 2003). The idea of electrically stimulating the scalp (to indirectly stimulate the brain) so as to examine its function was somewhat forgotten afterwards, but underwent a revival starting in the 1960s where the efficacy of tES, especially tDCS, in clinical settings was tested systematically (reviewed in Priori, 2003). Results indicated an improvement in several pathological conditions, mainly in depression (e.g. Costain, Redfearn, & Lippold, 1964; Redfearn, Lippold, & Costain, 1964), and partly in schizophrenia (Herjanic & Moss-Herjanic, 1967; but see Lifshitz & Harper, 1968).

The recent revival of interest in tES methods largely began from the use of tDCS as a tool to modulate cortical excitability in the motor system (Nitsche & Paulus, 2000; Priori, Berardelli, Rona, Accornero, & Manfredi, 1998). In parallel, however, tDCS experienced a steady increase in popularity for clinical research (e.g. Flöel, 2014; Miniussi et al., 2008). Finally, in the last decade, tES has found its way into more basic research, inspired by reports that tES can impact on memory (e.g. Fregni et al., 2005), attention (Moos, Vossel, Weidner, Sparing, & Fink, 2012), perception (e.g. Kanai, Chaieb, Antal, Walsh, & Paulus, 2008), or motor skills (e.g. Pollok, Boysen, & Krause, 2015), although critical voices have also been raised (Horvath, Forte, & Carter, 2015a, 2015b; Iuculano & Cohen Kadosh, 2013).

When systematic experimental approaches for the use of tES in speech research started less than 10 years ago, they focused on resolving difficulties in speech production instead of perception or comprehension. tES turned out to be a promising tool for language therapy and important work suggested tDCS an appropriate method for the treatment of aphasia (e.g. Fridriksson, Richardson, Baker, & Rorden, 2011). The potential of tDCS for improving performance in naming tasks has been repeatedly demonstrated (e.g. Holland et al., 2011; Monti et al., 2013) and there are already several comprehensive reviews of this literature (Fridriksson, Hubbard, & Hudspeth, 2012; Málly, 2013). In this review, we will instead concentrate on those studies that tested the impact of tES on speech perception or comprehension. This is a very recent line of research: the bulk of the available literature was published in the last five years and most of these studies used tDCS (rather than tACS) as a tool for the investigation of speech perception or comprehension. Given that tACS is under-explored (only two studies of speech processing reported, to-date), we have therefore also included example studies that reported effects of tACS on basic auditory processing rather than speech processing. These illustrate the potential and promise for showing tACS effects on auditory perception (for a review, see Heimrath, Fiene, Rufener, & Zaehle, 2016). However, we acknowledge that more work needs to be done to apply similar methods to the investigation of speech perception and comprehension.

## Overview of the method

**Figure 1.**
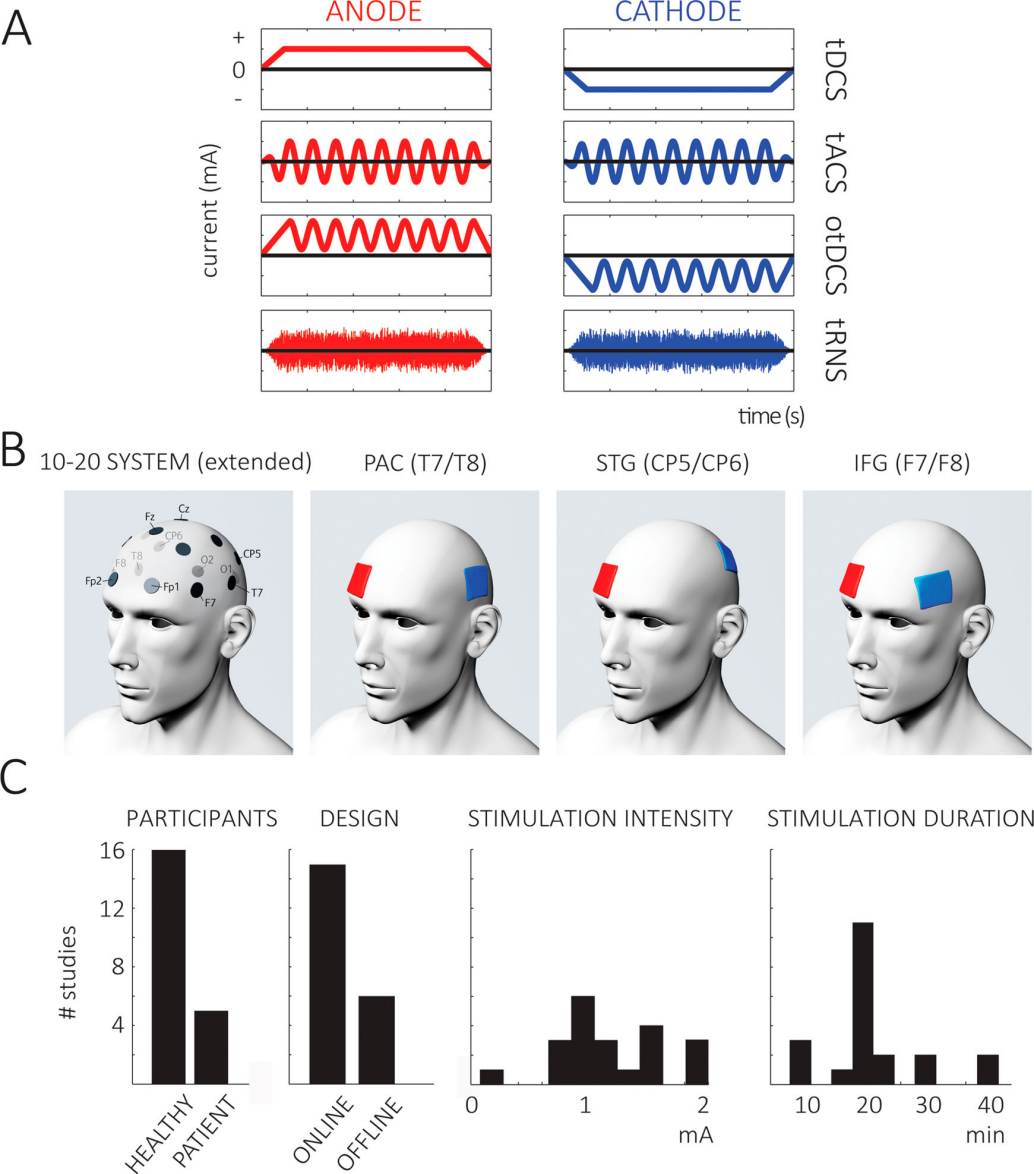
(A) Current waveforms used for stimulation during the different types of tES. In addition to the relatively established tDCS and tACS methods (first and second row, respectively), these methods can be combined (oscillatory tDCS, otDCS, third row), or a noise with a broad spectral range can be applied (tRNS, fourth row). At the start (and end) of stimulation, current is commonly faded in (and out) to minimise sensations associated with stimulation. Note the opposite sign of the current for anode (left) and cathode (right). Redrawn, with modifications, from Herrmann et al. (2013) and Saiote et al. (2013). (B) Electrodes are commonly placed above the to-be-stimulated region. This panel shows these placements (according to the extended 10–20 system, chosen electrodes are shown in the leftmost panel) for the three groups of studies described in this review (T7/T8, above PAC; Cp5/Cp6, above STG/STS; F7/F8, above IFG; for IFG, other electrode positions have been described). Blue (cathodal) electrodes are shown exemplarily above target regions of the left hemisphere, and the positions of the red (anodal) “return” electrodes are shown as attached to the transorbital region of the contralateral hemisphere (although this approach is commonly found in the literature, it is a rather arbitrary decision, see text for discussion of associated problems). In practice, the position of the “return” electrode (e.g. the anode for cathodal stimulation), the hemisphere (left, right, or both) for the “stimulation” electrode (e.g. the cathode for cathodal stimulation), and the direction of current flow (cathodal, anodal, or oscillatory) varies across studies. (C) Number of different studies (out of the 21 studies reviewed in detail in the section “Key empirical contributions”) which employ different participants, experimental designs (left two graphs), and stimulation parameters (right two graphs). The diversity of these parameters across studies makes comparisons difficult.

In tES, a weak (direct or alternating; Figure 1(A)) current is applied to the scalp, a small fraction of which can reach neural tissue and influence neural processing (Nitsche & Paulus, 2000). The simplest setup for a tES experiment consists of a stimulator box plus two electrodes: a positively charged “anode” and a negatively charged “cathode”. In tDCS, “anodal” or “cathodal” stimulation commonly refers to the anode or cathode placed above or close to a brain region that is the target of stimulation, respectively. The other electrode (cathode or anode, respectively, sometimes called “reference” or “return” electrode) is often (and rather arbitrarily) placed above a brain or body region assumed not to be directly involved in the task (as shown in Figure 1(B)). In tACS, the direction of current flow between the two electrodes alternates periodically. Although relatively weak, tES can produce noticeable sensations (e.g. prickling on the skin; Fertonani, Ferrari, & Miniussi, 2015). Therefore, if a change in behavioural outcome (or other dependent measures, such as electrophysiological signals) induced by tES is described in the literature, this commonly refers to a comparison with a sham stimulation group in which similar sensations were produced by ramping up and down the electrical current.

Whereas the magnetic pulse administered in tMS can directly *generate* action potentials in neurons within stimulated regions, tES rather affects the *likelihood* of action potentials by changing the ionic gradient across neuronal membranes (Herrmann et al., 2013; Purpura & Mcmurtry, 1965; Thut, Schyns, & Gross, 2011) during stimulation (i.e. *online*). Additional effects *after* application of tES (i.e. *offline* effects) have also been described (see below). During stimulation, tDCS results in a shift in neuronal membrane potentials; this was demonstrated by showing that motor potentials evoked by tMS (Adank et al., in press) are modulated by tDCS. In earlier studies, it was shown that the direction of this modulation of motor potentials seems to depend on the direction of current flow: an anodal electrode above the target site led to an increased motor potential, whereas a cathodal stimulation led to a decreased motor potential (Nitsche & Paulus, 2000; Priori, 2003; Priori et al., 1998). These results have been widely cited as suggesting that anodal tDCS is “excitatory” and cathodal tDCS is “inhibitory” based on their effect on neuronal excitability (e.g. Nitsche & Paulus, 2000). However, recent studies testing different stimulation parameters or that aim at extending this evidence to areas beyond the motor system did not confirm this conclusion. Instead, there seems to be a complex interplay between stimulation site, duration, and task that determines whether a given tDCS protocol increases, decreases, or does not affect neuronal excitability (Bestmann, de Berker, & Bonaiuto, 2015; Parkin, Ekhtiari, & Walsh, 2015). Indeed, even for the motor system, increasing the duration or intensity of stimulation can turn neuronal excitation into inhibition (Batsikadze, Moliadze, Paulus, Kuo, & Nitsche, 2013; Monte-Silva et al., 2013). This issue leads to greater complexity in comparing the results of different studies (see the section below on “Challenges for studying spoken language”) and to a need for further studies examining the neuronal consequences of tES more directly. Aftereffects induced by one session of tDCS can last up to 90 minutes and are commonly assumed to reflect long-term potentiation (LTP) or depression (LTD) at the synaptic level (e.g. Nitsche & Paulus, 2001; Priori, 2003; Stagg & Nitsche, 2011).

It is commonly assumed that the application of the periodic current in tACS (Figure 1(A), second row) directly influences neural oscillations, by inducing rhythmic changes in neuronal excitability (Buzsáki & Draguhn, 2004), in a frequency range that corresponds to the frequency of stimulation. Indeed, an impact of tACS on brain oscillations has been demonstrated in intracranial (Ozen et al., 2010) and electro- or magnetoencephalographic (EEG/MEG; Helfrich et al., 2014; Neuling et al., 2015) recordings and even indirectly using functional magnetic resonance imaging (fMRI; Vosskuhl, Huster, & Herrmann, 2015a). Notably, whereas neuronal firing depends on the phase of neural oscillations in the absence of stimulation, it can be made to depend on the phase imposed by external electrical stimulation (Fröhlich & McCormick, 2010; Ozen et al., 2010). This result suggests that tACS modulates neuronal excitability in a rhythmic fashion, and that neural oscillations can be entrained by means of tACS (for a review, see Thut et al., 2011). Electrophysiological aftereffects lasting up to 30 minutes can be induced by tACS (Neuling, Rach, & Herrmann, 2013; summarised by Veniero, Vossen, Gross, & Thut, 2015); this may be consistent with synaptic changes (Vossen, Gross, & Thut, 2015), or due to lasting hyperpolarisation of membrane potentials.

## Challenges for studying spoken language

Most of the challenges described here do not apply exclusively to the investigation of speech processing, but are generic to any (relatively) newly established method where experimental protocols have not yet been standardised. In a later section “Future directions”, we discuss potential solutions to the challenges presented here.

### Comparability across studies

There are, as yet, no universally accepted stimulation parameters for tES. As we allude to in our review of the existing literature below and summarise in Figure 1(C) and Tables 1–3, studies applying tES for the investigation of speech processing differ widely in key parameters such as (1) stimulation intensity or duration, (2) electrode location, (3) stimulation protocol, and (4) task. It has recently been pointed out that changes to these parameters can lead to very different, and even opposite, outcomes (Antal, Keeser, Priori, Padberg, & Nitsche, 2015; Monte-Silva et al., 2013; Parkin et al., 2015). Similar points have been made for the comparison of online and offline stimulation which can result in different outcomes even for identical stimulation parameters (Pirulli, Fertonani, & Miniussi, 2013). Furthermore, it is possible that individual participants’ “brain state” during stimulation (e.g. the state of neural excitability in the case of tDCS, or the amount of instantaneous power in oscillatory frequency bands in the case of tACS) might impact on observed outcomes (Neuling et al., 2013). Some studies reported effects of tES only if the target region was activated by a task that involved neural processing in the respective brain region (Antal, Polania, Schmidt-Samoa, Dechent, & Paulus, 2011; Vosskuhl et al., 2015a). Accordingly, results can be very difficult – or even impossible – to compare between studies.

**Table 1.** Summary of studies using tES assumed to target PAC for the investigation of speech and non-speech auditory perception (non-speech studies are marked with an asterisk).

Study	population	*N*	Method	“Active” electrode	“Reference” electrode	Protocol	Timing	Outcome	Control
Marques et al. (2014)	Healthy	24	tDCS	T3/T4 (corr. to T7/T8)	Right deltoid muscle	20 min. of 2 mA	Online	Bilateral cathodal (but not anodal) tDCS decreases McGurk effect	Sham (same subjects)
Heimrath, Fischer, et al. 2016	Healthy	13	tDCS	T7/T8	Cz	22 min. of 1.5 mA	Online	Bilateral cathodal (but not anodal) tDCS improves phonetic categorisation	Sham (same subjects)
Neuling, Rach, Wagner, Wolters, and Herrmann (2012)*	Healthy	16	otDCS	T7/T8	n/a	DC of 1 mA + 10 Hz AC of 0.425 mA (mean), 2 × 21 min.	Online	Detection of non-speech target tones embedded in noise depends on phase of the imposed current	No control
Riecke, Formisano, et al. (2015)*	Healthy	14	tACS	T7/T8	Cz	4 Hz, 0.8 mA (mean), 39.6 min.	Online	Detection of click trains depends on phase of the imposed current	Sham (same subjects)
Riecke, Sack, et al. (2015)*	Healthy	20	tACS	T7/T8	Cz	4 Hz, 0.8 mA (mean), 40 min.	Online	Time required to segregate non-speech target sounds from background sounds depends on tACS phase	Sham (same subjects)
Rufener, Zaehle, et al. (2016)	Healthy	21 (+17 for no stim.)	tACS	T7/T8	n/a	40 Hz, 1.1 mA (mean), 18 min.	Offline	40-Hz (but not 6-Hz) tACS impairs learning performance in phonetic categorisation task	No stimulation, control frequency
Rufener, Oechslin, et al. (2016)	Healthy	25	tACS	T7/T8	n/a	40 Hz, 1.38 mA (mean), 8 min.	Online	Replicate previous study and show that results are reversed for older listeners	Control frequency

Note: Stimulation methods (tDCS/tACS/otDCS and electrode locations) are illustrated in Figure 1.

**Table 2.** Summary of studies assumed to target STG/STS.

Study	Population	*N*	Method	“Active” electrode	“Reference” electrode	Protocol	Timing	Outcome	Control
Flöel et al. (2008)	Healthy	19	tDCS	Cp5	Contralateral supraorbital area	20 min. of 1 mA	Online	Anodal (but not cathodal) tDCS over left STG improves associative learning of visual words and auditory pseudowords	Sham (same subjects)
Meinzer et al. (2014)	Healthy	20 (+20 for sham)	tDCS	Cp5	Contralateral supraorbital area	5 × 20 min. of 1 mA	Online	Anodal tDCS over left STG improves associative learning of visual pseudowords and pictures	Sham
Savill et al. (2015)	Healthy	24	tDCS	Cp5	Contralateral supraorbital area	15 min. of 1.5 mA	Online	Anodal tDCS over left STG during acquisition of spoken pseudowords improves performance in learning task on the next day	Sham (same subjects)
Peretz & Lavidor (2013)	Healthy	17	tDCS	Cp5/Cp6 (separate sessions)	Contralateral orbitofrontal cortex	10 min. of 1 mA	Offline	Anodal tDCS over right STG decreases reaction time in lexical ambiguity task (only for subordinate associations)	Sham (same subjects)
Price et al. (2016) †	Healthy	18	HD-tDCS	Cp5/C6 (separate sessions)	Ring of four electrodes, ∼6 cm away from target electrode	21 min. of 2 mA	Online	Anodal tDCS over left (but not right) AG increases processing speed of written semantic information	Sham (same subjects)
Wang et al. (2013)	Aphasia	1	tDCS	Cp5/Fc3 (separate sessions)	Contralateral shoulder	10 × 20 min. of 1.2 mA	Offline	Anodal tDCS over left STG and left IFG improves auditory word identification after five sessions (no further improvement after 10 sessions)	Sham (same subject)
Wu et al. (2015)	Aphasia	12	tDCS	Cp5	Contralateral shoulder	5 × 20 min. of 1.2 mA	Offline	Anodal tDCS over left STG improves auditory word identification	Sham (same subjects)
You et al. (2011)	Aphasia	7 (+7 for sham)	tDCS	Cp5/Cp6 (separate sessions)	Contralateral supraorbital area	10 × 30 min. of 2 mA	Offline	Cathodal tDCS over right STG (but not anodal tDCS over left STG) improves auditory verbal comprehension	Sham
Riedel et al. (2015)	Healthy	17 (+17 for sham; +17 for contr)	tDCS	Det. by MRI/neuro-navigation	Contralateral orbit	20 min. of 0.75 mA	Online	Cathodal tDCS over left pSTS (but not over BA6/44) decreases visual and auditory speech recognition	Sham, control location

Note: Studies with a slightly different target site are marked with a cross. Organisation same as in Table 1.

**Table 3.** Summary of studies assumed to target IFG.

Study	Population	*N*	Method	“Active” electrode	“Reference” electrode	Protocol	Timing	Outcome	Control
Lupyan et al. (2012)	Healthy	20 (+20 for contr)	tDCS	F7	Contralateral mastoid	20 min. of 1.5 mA	Online	Cathodal tDCS over left IFG leads to poorer semantic categorisation	Control (no details)
Alexander et al. (2012)	Healthy	13	tDCS	Fc4	Contralateral frontopolar cortex	10 min. of 1 mA	Offline	Cathodal (but not anodal) tDCS over right IFG improves prosody comprehension	Sham (same subjects)
Sehm et al. (2013)	Healthy	12 (+12 for sham; +12 for contr.)	tDCS	Det. by MRI	Contralateral supraorbital area	3 × 20 min. of 1 mA	Online	Anodal tDCS over left IFG (but not over left IPC) improves perceptual learning of degraded words	Sham, control location
Wang et al. (2013)	Aphasia	1	tDCS	Cp5/Fc3 (separate sessions)	Contralateral shoulder	10 × 20 min. of 1.2 mA	Offline	Anodal tDCS over left STG and left IFG improves auditory word identification after five sessions (no further improvement after 10 sessions)	Sham (same subject)
Pinchuk et al. (2015)	Children with disorders of psych. develop.	26 (+10 for no stim.)	tDCS	Not precisely defined	Ipsilateral mastoid	5–9 × 25–35 min. of 0.06–0.09 mA	Offline	Anodal tDCS over left IFG or “left temporo-parieto-occipital area” brings laterality index during dichotic listening of children with disorders of speech and language closer to that of healthy children	No stimulation
Schaal et al. (2015)*†	Congenital amusia	9 (+8 for no stim.)	tACS	Det. by neuro-navigation	Contralateral supraorbital area	35 Hz, 1 mA, max. 20 min.	Online	35-Hz (but not 90-Hz) tACS over right DLPFC improves pitch memory	No stimulation, control frequency

Note: Organisation same as in Tables 1 and 2.

### Electrode montage

Most studies target their region of interest by placing one of the stimulation electrodes directly above that brain region (blue electrodes in Figure 1(B)) – however, recent modelling studies suggested that a complicated interaction between electrode shape, orientation, material, and the individual properties of the stimulated brain tissue takes place (Saturnino, Antunes, & Thielscher, 2015). In combination, this can result in current flow, and hence effects on neuronal excitability, that might be completely different from those expected. It has been reported that the current flow might even be maximal *between* electrodes (Antal et al., 2014; Rampersad et al., 2014), suggesting a more important role of the “reference” electrode (e.g. the anode in the case of cathodal stimulation; red electrodes in Figure 1(B)) than previously thought. Indeed, if one electrode is placed over occipital cortex while the position of the other (“reference”) electrode is altered, this can abolish the efficacy of tDCS in modulating the visually evoked potential (VEP) or even lead to opposite results (Accornero, Li Voti, La Riccia, & Gregori, 2007; Antal et al., 2015; Antal, Kincses, Nitsche, Bartfai, & Paulus, 2004). This shows that not only the polarity of the electrodes affects neuronal excitability (here reflected in the VEP), but also their position. It also provides a further reason why the simple assumption that anodal and cathodal stimulation always results in increased and decreased neuronal excitability, respectively, is too simplistic. Additionally, the current flow introduced by tES may be spatially unspecific and cover a broad range of brain regions (Neuling, Wagner, Wolters, Zaehle, & Herrmann, 2012; Nitsche et al., 2007). Consequently, targeting specific brain areas that are critical for speech processing is not straightforward.

### Impact of tES on cognitive and neurophysiological measures

Part of the problem for establishing the replicability of tES is the need to choose outcome measures that reflect specific underlying processes involved in speech perception and comprehension and that can be reliably perturbed by tES. tES was reported to have an impact on the neural response evoked by a transient event, such as by a simple visual or auditory stimulus (Accornero et al., 2007; Zaehle, Beretta, Jäncke, Herrmann, & Sandmann, 2011). However, speech is a continuous auditory stimulus, such that electrophysiological responses are more complex than those seen for punctate auditory or visual stimuli (see also Wöstmann, Fiedler, & Obleser, in press). Moreover, in contrast to speech production, where picture naming is an established task for which speed and accuracy can be readily measured (for a review, see Monti et al., 2013), there is a very wide range of tasks that have been proposed to provide a similar measure for speech perception or comprehension (see the extensive review volume coordinated by Grosjean & Frauenfelder, 1996, for details). As reviewed below, very few of these tasks have thus far been explored with tES and systematic comparisons of different tasks and stimulation parameters are desperately needed. Indeed, in meta-analyses, Horvath and colleagues were unable to find a statistically significant impact of tDCS on any cognitive (Horvath, Forte, & Carter, 2015b) or electrophysiological measures (Horvath et al., 2015a) other than modulation of the tMS-evoked motor potential. However, this negative finding is currently debated and has to be interpreted carefully: in particular, the provided findings might be explained by the diversity of key stimulation parameters used in reported experiments (see point 1) which makes pooling across studies difficult or even inappropriate (Antal et al., 2015). Moreover, the division into specific cognitive sub-domains by Horvath and colleagues resulted in a very small number of studies per analysis, reducing the reliability of its outcome and making interpretation difficult. Indeed, for speech output measures (mostly verbal fluency), and for (mostly) frontal stimulation, a recent meta-analysis of eight studies showed a reliable effect of a single tDCS session on the accuracy of speech production (Price, McAdams, Grossman, & Hamilton, 2015). Importantly, these eight studies were also included in the meta-analysis by Horvath et al. (2015b), suggesting that differences in meta-analysis methods can change the conclusions drawn. Further, more focussed, meta-analyses would be a valuable addition to the literature.

### Speech-specific effects of tES

Due to the spatial unspecificity of tES, it is unlikely that the stimulation will only affect neural activity in a single brain area. Thus, even if stimulation successfully targeted a region that specifically contributed to the processing of speech sounds, additional stimulation of adjacent less-specific regions could not be excluded. It is thus difficult to distinguish acoustic from speech-specific effects of stimulation: improved speech comprehension during tES, for instance, could be (1) due to a specific enhancement of speech processing, (2) changes to hearing thresholds that would affect a large variety of acoustic stimuli, or (3) even a reflection of muscle stimulation in the ear (Zoefel & Heil, 2013). In studies using tES for speech research, it is thus critical to have experimental control conditions in which these other effects can be disentangled: for instance, if behavioural consequences of tES are stronger for speech stimuli or tasks than for non-speech equivalents, it might be appropriate to assume a speech-specific effect of stimulation.

## Key empirical contributions

In the following section, we summarise published studies that use tES as a tool for the investigation of speech perception and comprehension. We have grouped these based on the scalp (and presumed neural) location of stimulation (Figure 1(B)). Experimental details are provided in tables (one for each location).

## Primary Auditory Cortex (PAC): T7/T8 (extended 10–20 system)

Two published studies have reported effects of tDCS applied over PAC on speech perception. Marques, Lapenta, Merabet, Bolognini, and Boggio (2014) found that cathodal (but not anodal) tDCS can decrease the McGurk illusion (McGurk & MacDonald, 1976) in which the combined presentation of an auditory (e.g. /ba/) and a different visual syllable (e.g. /ga/) leads to the percept of an intermediate version (e.g. /da/). Cathodal (but not anodal) tDCS over PAC was also shown to improve the identification of syllables (e.g. /ta/ vs. /da/) in a study by Heimrath, Fischer, Heinze, and Zaehle (2016).

It has also been shown that tACS can influence activity in PAC, and that this stimulation has perceptual and behavioural consequences for non-speech stimuli: Neuling, Rach, et al. (2012) showed that detection of a pure tone at the threshold level depends on the phase of oscillating tDCS at 10 Hz (otDCS, see Figure 1(A), third row; since this includes oscillatory stimulation we will group this method with other tACS studies). Their behavioural findings were supported by their use of anatomical modelling of current flow so that Neuling et al. were able to infer that the neural currents produced by their stimulation protocol were likely to be maximal in PAC. Similar behavioural findings, using bilateral 4-Hz tACS and click trains, were presented by Riecke, Formisano, Herrmann, and Sack (2015). Moreover, it was demonstrated in a later study (Riecke, Sack, & Schroeder, 2015) that the phase of the 4-Hz tACS current determines the time needed to detect a target sequence in background noise. Two studies investigated modulation of speech perception by means of tACS applied to PAC: using similar stimuli as in the Heimrath, Fischer, et al. (2016) tDCS study described above, Rufener, Zaehle, Oechslin, and Meyer (2016) showed that perceptual learning of a phonetic categorisation task (e.g. /ta/ vs. /da/) is impaired in young participants by 40 Hz, but not by 6-Hz tACS. The authors replicated this impairment in a second study and were able to show (surprisingly) that tACS at 40 Hz can improve performance in the same task if it is applied in older listeners (Rufener, Oechslin, Zaehle, & Meyer, 2016).

Together, these studies suggest that both cathodal tDCS and tACS involving positions T7 and T8 of the extended 10–20 system might be an effective tool to study auditory contributions to speech perception, potentially by influencing neural activity in general (in the case of tDCS) or neural oscillations (in the case of tACS) in these regions.

## Posterior Superior Temporal Gyrus/Sulcus (STG/STS): Cp5/Cp6, P7

It is a relatively consistent finding that anodal tDCS over Posterior Superior Temporal Gyrus (STG) has an impact on speech processing. Flöel, Rösser, Michka, Knecht, and Breitenstein (2008) showed improved associations between spoken pseudowords and visual stimuli when anodal tDCS was applied during learning. This finding has since been replicated for participants acquiring associations between written pseudowords and pictures (Meinzer et al., 2014), and extended by Savill et al. (2015), who reported improved performance on the next day when sequences of previously acquired spoken pseudowords had to be memorised and recalled without their corresponding visual stimuli. Anodal tDCS of right STG also decreased response time in a task where subjects had to judge whether a written word (e.g. “farmer”) was related to the subordinate meaning of a lexically ambiguous word (e.g. “pen”; Peretz & Lavidor, 2013). Although not all of these studies directly measured speech *perception,* they nevertheless used tES to provide evidence for the importance of STG for higher-level aspects of language processing (phonological learning and semantic access) that we might anticipate to involve shared systems for spoken and written language. One further possibility, however, is that effects of stimulation also extended to adjacent inferior parietal regions such as the Angular Gyrus (AG): a recent high-definition tDCS study (using multiple ring electrodes to produce more focal stimulation) showed effects of AG stimulation on combinatorial semantic processing of written words (Price, Peelle, Bonner, Grossman, & Hamilton, 2016).

In patients with different types of aphasia (primarily impairment of speech production, though including more mixed profiles), auditory word comprehension was shown to be improved by anodal stimulation of the left STG (Wang, Wu, Chen, Yuan, & Zhang, 2013; Wu, Wang, & Yuan, 2015; note that the stimulation protocol in the former study also included stimulation of Inferior Frontal Gyrus (IFG) in different sessions). The effect of anodal tDCS over the left STG on auditory verbal comprehension was not confirmed, however, in other data published by You, Kim, Chun, Jung, and Park (2011); instead, they found an improved performance when cathodal tDCS was applied over the right STG. It is possible that this finding reflects suppression of aberrant contralateral activity. In line with the proposal that cathodal stimulation can impair processing, Riedel, Ragert, Schelinski, Kiebel, and von Kriegstein (2015) showed a decline in the recognition or auditory (and visual) speech induced by cathodal tDCS over left Superior Temporal Sulcus (STS).

In sum, stimulation of STG or STS using tDCS is both feasible and has produced promising results in initial studies. Nevertheless, results are not entirely consistent and further replications – including studies that delimit the tasks and stimuli that specifically are, and are not, enhanced by tDCS – would be helpful. At present, we are not aware of published demonstrations of the effectiveness of tACS to target these regions.

## IFG: F7/F8, Fc3/Fc4

In comparison to the relatively consistent results reported for PAC stimulation and mixed effects of STG stimulation, even less consistent results have been reported for the application of tDCS over IFG during receptive language tasks. For example, Lupyan, Mirman, Hamilton, and Thompson-Schill (2012) and Alexander, Avirame, and Lavidor (2012) applied cathodal tDCS to left and right IFG, respectively, but semantic categorisation declined in the former whereas prosody comprehension was improved in the latter study (anodal stimulation did not result in reliable effects). Sehm et al. (2013) did not test cathodal stimulation, but reported improved perceptual learning of degraded (vocoded) spoken words when anodal tDCS was applied to left IFG during training sessions conducted over several days. In the clinical case study mentioned above (Wang et al., 2013), a combination of anodal STG and IFG stimulation (in different sessions) resulted in improved auditory word comprehension. Finally, in a study of developmental language-impaired individuals, the functional asymmetry typically present during dichotic listening (i.e. which ear is dominant when reporting simultaneously presented dichotic syllables) was partly restored to normal levels by means of anodal tDCS over left IFG (Pinchuk, Wasserman, Wasserman, Sirbiladze, & Kartashev, 2015; note that this study also included subjects stimulated in other, vaguely defined, areas in the left hemisphere). Again, we are not aware of published studies using tACS to target IFG. However, a non-speech study by Schaal, Pfeifer, Krause, and Pollok (2015) is worth mentioning, in which tACS at 35 Hz but not 90 Hz over right dorsolateral prefrontal cortex improved pitch memory in individuals with congenital amusia. Similar studies with linguistic materials could be informative.

As we have seen, the results of these studies are rather inconsistent, but also the specific experimental and stimulation parameters vary largely. This might be an important reason why it is difficult to combine them to formulate a general conclusion about the effectiveness of tES for investigating the role of IFG in receptive speech processing despite clear evidence for an impact of frontal tDCS on speech production (as shown in the meta-analysis by Price et al., 2015). Clearly, more research is needed here, including publication of null or negative findings. Only by careful consideration of successful and null findings can we determine which forms of stimulation, and which functions, can be supported or disrupted by tES of inferior frontal regions.

## Future directions

The diversity of stimulation and experimental parameters (cf. Figure 1(C) and Tables 1–3) complicates the comparison across studies available in the literature. Perhaps reassuringly, in the results described above, tES above target regions for which stimulation parameters were kept relatively consistent across studies (e.g. PAC) led to relatively homogeneous results whereas stimulation of regions with more variable stimulation parameters (e.g. IFG) resulted in less clear and consistent findings. These observations suggest a need to standardise stimulation and other experimental parameters (e.g. appropriate measures for speech perception or comprehension) for future studies (e.g. Nitsche et al., 2008; Parkin et al., 2015; Woods et al., 2016). In particular, consensus has to be found with respect to the optimal location of electrodes to target a given brain region. Our review of the existing literature suggests that stimulation over T7 and T8 (i.e. above PAC) seems to be the electrode montage that produces the most consistent and reliable effects for the investigation of speech perception or comprehension. This might seem surprising at first glance, as brain regions further up the auditory hierarchy (e.g. in the STG), are suggested by functional imaging to make a more specific contribution to the processing of speech (McGettigan & Evans, in press). However, based on the challenges described above, we cannot rule out the possibility that this electrode montage also stimulates STG or other speech-specific regions, instead of or in addition to PAC. This view is supported by results obtained in a recent modelling study; based on magnetic resonance images, Opitz, Paulus, Will, Antunes, and Thielscher (2015) developed head models for two subjects and systematically simulated the effects of different anatomical factors (e.g. skull thickness and composition) on current flow during electrical stimulation. Importantly, results indicated that the current flow introduced by tES is negatively correlated with sulcal depth (i.e. reduced current flow further away from the cortical surface). Thus, given the position of PAC (deep in the lateral sulcus), this might reduce the effect of stimulation to some degree while stimulation of more superficial regions (such as lateral STG) cannot be ruled out. An interesting solution, leading to an increased focality of the stimulation, has been proposed recently (Datta et al., 2009; Edwards et al., 2013). Here, several small round electrodes are arranged in a circular fashion (“ring”) around a single electrode above the target region, which is of the same size and shape but opposite polarity. This method has been used to good effect in stimulating the AG (Price et al., 2016).

A crucial step forward has been made in the last years by introducing the possibility to combine tES with imaging methods, such as EEG/MEG or fMRI (Herrmann, Strüber, Helfrich, & Engel, 2016; Saiote, Turi, Paulus, & Antal, 2013). A combination of these methods opens up a wider range of physiological variables that can be measured *during* stimulation, such as the entrainment of neural oscillations (Helfrich et al., 2014; Neuling et al., 2015) or the blood oxygen-level dependent (BOLD) response (Antal et al., 2011; Vosskuhl et al., 2015a). Critically, these combined recordings can provide additional evidence for whether a given electrode configuration or stimulation protocol for tES can:

- change neural activity (as measured in the BOLD response) in brain regions we want to target, but not in brain regions we do not want to target (using tES-fMRI).
- change neural reactivity in response to stimulus input (i.e. the evoked response) in the modality we want to target (see Zaehle et al., 2011, for an example concerning the auditory domain), but not in modalities we do not want to target (using tES-EEG/MEG).
- entrain neural oscillations in frequency bands we want to entrain, without influencing them in frequency bands we do not want to influence (using tACS-EEG/MEG).

To conclude, these exciting new developments make it all the more urgent that systematic studies be conducted to investigate the effect of different stimulation parameters (Opitz et al., 2015; Saturnino et al., 2015), combined with modelling of the current flow during stimulation (Neuling, Wagner, et al., 2012; Ruffini, Fox, Ripolles, Miranda, & Pascual-Leone, 2014) and/or approaches in which superficial stimulation is coupled with intracranial recordings (Ozen et al., 2010).

The number of studies using tACS in speech research is very low. This is somewhat surprising given the hypothesised role for neural oscillations in the processing of speech. For instance, the alignment between neural oscillations and the rhythm of speech (∼4 Hz; Zoefel & VanRullen, 2015a, 2015b) is associated with an improved speech comprehension (Luo & Poeppel, 2007; Peelle, Gross, & Davis, 2013; for a review, see Zoefel & VanRullen, 2015c) and alpha oscillations (∼10 Hz) seem to be important for speech processing (Strauß, Wöstmann, & Obleser, 2014; Wöstmann, Herrmann, Wilsch, & Obleser, 2015). Thus, the application of tACS at 4 and/or 10 Hz accompanied by measurement of the behavioural and physiological consequences for speech processing is an interesting approach for future experiments.

Finally, other promising variants of tES have been developed in recent years whose application for speech research has not been tested yet but might be worth trying (Figure 1(A)). A combination of tDCS and tACS is called oscillatory tDCS (otDCS) in which alternating current is applied with an additional DC offset, assumedly modulating neural oscillations and excitability at the same time (Herrmann et al., 2013; Veniero et al., 2015). otDCS is commonly applied in sleep research (Marshall, Helgadóttir, Mölle, & Born, 2006) but has been successfully introduced into auditory research as already outlined above (Neuling, Rach, et al., 2012). Transcranial random noise stimulation (tRNS) is related to tACS and is also hypothesised to affect neural oscillations; however, the frequency of stimulation changes continuously and randomly in a broad range of frequencies (Paulus, 2011) which is proposed to increase neural excitability (Terney, Chaieb, Moliadze, Antal, & Paulus, 2008). The question of whether tDCS, tACS, or other variants of tES is the method of choice for modulating speech perception and comprehension remains to be explored. There are few direct comparisons of these different methods in the published literature.

## Conclusion

We conclude this review by highlighting tES as a tool in speech research that shows great promise, despite several challenges that remain to be solved. Many of these issues are due to the fact that tES is a relatively new method and standardisation of experimental protocols requires time. Once these challenges have been overcome, however, we anticipate that tES will become a popular tool to investigate the causal role of brain activity for the perception and comprehension of speech.
